# Clusters of medical specialties around patients with multimorbidity – employing fuzzy c-means clustering to explore multidisciplinary collaboration

**DOI:** 10.1186/s12913-023-09961-z

**Published:** 2023-09-09

**Authors:** Marlies Verhoeff, Liann I. Weil, Hung Chu, Yolande Vermeeren, Janke de Groot, Jako S. Burgers, Patrick P. T. Jeurissen, Leslie R. Zwerwer, Barbara C. van Munster

**Affiliations:** 1 Department of Geriatric Medicine, University Medical Center Groningen, University of Groningen, Groningen, the Netherlands; 2 Knowledge Institute of the Federation of Medical Specialists, Utrecht, the Netherlands; 3 Donald Smits Center for Information and Technology, University of Groningen, Groningen, the Netherlands; 4 Department of Internal Medicine, Gelre Hospitals, Apeldoorn/ Zutphen, the Netherlands; 5 Maastricht University, Care and Public Health Research Institute (CAPHRI), Maastricht, the Netherlands; 6 Scientific Center for Quality of Healthcare (IQ healthcare), Radboud Institute for Health Sciences, Radboud University Medical Center, Nijmegen, the Netherlands; 7 Department of Health Sciences, University Medical Center Groningen, University of Groningen, Groningen, the Netherlands

**Keywords:** Multimorbidity, Care coordination, Machine learning, Multidisciplinary collaboration, Cluster analysis, Electronic health records

## Abstract

**Background:**

Hospital care organization, structured around medical specialties and focused on the separate treatment of individual organ systems, is challenged by the increasing prevalence of multimorbidity. To support the hospitals’ realization of multidisciplinary care, we hypothesized that using machine learning on clinical data helps to identify groups of medical specialties who are simultaneously involved in hospital care for patients with multimorbidity.

**Methods:**

We conducted a cross-sectional study of patients in a Dutch general hospital and used a fuzzy c-means clustering algorithm for the analysis. We explored the patients’ membership degrees in each cluster to identify subgroups of medical specialties that provide care to the same patients with multimorbidity. We used retrospectively collected electronic health record data from 2017. We extracted data from 22,133 patients aged ≥18 years who had received outpatient clinical care for two or more chronic and/ or oncological diagnoses.

**Results:**

We found six clusters of medical specialties and identified 22 subgroups. The clusters were labeled based on the specialties that most characterized them: 1. dermatology/ plastic surgery, 2. six specialties (gynecology/ rheumatology/ orthopedic surgery/ urology/ gastroenterology/ otorhinolaryngology), 3. pulmonology, 4. internal medicine/ cardiology/ geriatrics, 5. neurology/ physiatry (rehabilitation)/ anesthesiology, and 6. internal medicine. Most patients had a full or dominant membership to one of these clusters of medical specialties (11 subgroups), whereas fewer patients had a membership to two clusters. The prevalence of specific diagnosis groups, patient characteristics, and healthcare utilization differed between subgroups.

**Conclusion:**

Our study shows that clusters and subgroups of medical specialties simultaneously involved in hospital care for patients with multimorbidity can be identified with fuzzy c-means cluster analysis using clinical data. Clusters and subgroups differed regarding the involved medical specialties, diagnoses, patient characteristics, and healthcare utilization. With this strategy, hospitals and medical specialists can further analyze which subgroups are target populations that might benefit from improved multidisciplinary collaboration.

**Supplementary Information:**

The online version contains supplementary material available at 10.1186/s12913-023-09961-z.

## Background

The increasing prevalence of multimorbidity, generally defined as having two or more chronic diseases, challenges hospital care organization [1, 2]. The organization of hospital care is currently structured around medical specialties and focuses on the separate treatment of individual organ systems, which hinders care coordination for patients with multimorbidity [3, 4]. Consequently, patients with multimorbidity visit multiple healthcare professionals and have high healthcare utilization with a risk of interactions and contradictory treatments if multidisciplinary coordination is absent [1]. Moreover, multimorbidity is associated with worse health outcomes, increased burden of illness, limitations in function, and lower quality of life [5, 6].

However, organizational structures for multidisciplinary collaboration and coordination between medical specialists in the case of multimorbidity are limited. Organizing hospital care around specific patterns of multimorbidity or medical specialties might be helpful to facilitate multidisciplinary, coordinated care and prevent or minimize these outcomes. Cluster analysis of diseases is considered as an appropriate method to identify patterns of multiple diseases [7]. Several studies identified clusters of diseases, suggesting that underlying pathological processes might result in specific multimorbidity patterns [7–9]. Common identified patterns are cardiovascular and metabolic diseases, mental health-related problems, and musculoskeletal disorders. However, these disease patterns have been studied mostly outside of the hospital setting and primarily provide insights into the pathophysiology of multimorbidity.

Identifying clusters of medical specialties instead of clusters of diseases could support the hospitals’ realization of multidisciplinary care for patients with multimorbidity. Grouping patients with multimorbidity into clusters according to the simultaneous involvement of medical specialties might help identify potential target populations that can benefit from more multidisciplinary collaboration [1, 10]. This paper aims to identify clusters of medical specialties that are simultaneously involved in the hospital care for patients with multimorbidity, using machine learning and clinical data.

## Methods

### Data collection

For this retrospective, cross-sectional study, we retrieved all registered administrative EHR data from 2017 (01.01.2017 – 31.12.2017) of Gelre Hospitals in Apeldoorn, a middle-large teaching hospital in the Netherlands (number of beds = 542). The main variables of interest in the study were the medical specialties involved in a patient’s hospital care. Secondary variables included socio-demographics (age and sex), the diagnoses for which the patient had received hospital care, and the healthcare utilization per patient.

Variables were extracted from the hospital’s database, where the EHR data are stored for administrative and billing purposes. In the Netherlands, professionals record Diagnosis-Treatment Combinations (DTCs) to claim payments. Diagnosis-Treatment Combinations include data on the specialty involved per treatment, the diagnosis, and the number of care activities such as outpatient visits, emergency department (ED) visits, or inpatient days [11]. With the classification and definitions of the Dutch Hospital Data – Clinical Classification Software (DHD-CCS), all diagnoses in this study were classified into diagnosis types (acute, chronic, elective, oncological, and other). The DHD-CCS classification offers three different categorization levels, containing 233, 152, and 17 diagnosis groups. For this research, we used the level with 233 diagnosis groups (Supplementary file 1).

We included all adult patients (18+ years) who had received outpatient clinical care for two or more chronic diagnoses, two or more oncological diagnoses, or at least one chronic and one oncological diagnosis in 2017 (*n =* 22.133). The local ethics committee of Gelre Hospitals (Gelre LTC) approved the anonymous use of these data for this study and a waiver of informed consent (Gelre LTC number 2019_02). All methods were performed following relevant guidelines or regulations.

### Statistical analysis

Statistical analyses were performed using the free statistical software R (version 3.6.3), the fuzzy c-means clustering was performed using the packages “fclust” (version 2.1.1) and “ppclust” (version 1.1.0), and for the visualization we used the “ggplot2” package (version 3.3.3) [12–14].

Descriptive statistics were used to summarize demographic and healthcare characteristics. In addition, the frequencies and percentages of involved medical specialties and diseases were calculated.

### Fuzzy c-means clustering

With clustering, we aimed to group patients with multimorbidity according to the simultaneous involvement of medical specialties in their hospital care [15]. Cluster analyses assign patients to be as similar as possible within one cluster but as dissimilar as possible to individuals from other clusters. In fuzzy or soft clustering, in contrast to crisp or hard clustering, each patient is not assigned to one single cluster but a membership degree (i.e., membership), indicating the strength of a patient’s membership to each identified cluster [16]. Similar to previous studies of Marengoni et al. [17] and Violán et al. [18], we regarded the soft clustering method as more clinically meaningful than the hard clustering method because patients (the observations) would be able to be a member of more than one cluster of medical specialties. Therefore, we decided to employ the best-known soft clustering method: the fuzzy c-means algorithm [19].

With this clustering algorithm, patients were assigned membership degrees for clusters based on the simultaneous involvement of medical specialties in their hospital care. One medical specialty could be part of more than one cluster, considering all possible combinations of involved medical specialties. In addition, due to the fuzzy c-means algorithm, a patient could belong to several clusters of medical specialties, indicated by their membership to each identified cluster.

We used the clusters and the patients’ memberships for each cluster to identify subgroups of medical specialties that are simultaneously involved in the hospital care for patients with multimorbidity. The algorithm determines the common patterns of involved medical specialties, called clusters. It calculates for each patient to what extent they belong to or are ‘a member’ of the identified clusters, illustrated by the membership. For one patient (or observation), the sum of the memberships for all identified clusters is one [15]. The membership can be close to 0% (but never equal to 0%) if the patient’s pattern of involved medical specialties is most dissimilar to that specific cluster, or close to 100% (but never equal to 100%) if the patient’s pattern of involved medical specialties is most similar. Thus, in our applied fuzzy c-means clustering of medical specialties, patients can belong with a high membership to one specific cluster or combinations of clusters.

The main parameters of the fuzzy c-means clustering algorithm are the number of clusters (k) and the fuzziness parameter (m). If the fuzziness parameter m equals 1, the fuzzy c-means clustering is equivalent to k-means clustering. Whenever m is close to 1, memberships will be distributed towards one cluster over the others. Higher fuzziness parameters (e.g., in the limit of m to infinity) correspond to a fuzzy set of clusters, where memberships are more or less equally distributed across clusters [20]. We performed an exhaustive grid search of model hyperparameters for m=1.1, 1.2, 1.3, 1.4, 1.5, and k=5–15. That is, we examined each combination of the hyperparameters k and m. Since initial centroids in fuzzy c-means clustering are random, we performed 100 independent runs and subsequently selected the optimal run, which minimized the fuzzy c-means cost function. Finally, using the results of the optimal runs per hyperparameter combination, the optimal m and optimal k were identified using four validation indices: the Xie-Beni index, partition coefficient, partition entropy, and the Silhouette index [21–24]. Therefore, we first identified the optimal m value, corresponding to the minimum value for the Xie-Beni index and partition entropy, and the maximum value of the partition coefficient and Silhouette index. After choosing the optimal m value, all four indices were inspected with the chosen optimal m value for all tested k-values (k=5-15). The optimal number of clusters (k) was then determined based on the number for which the Xie-Beni and partition entropy had the lowest value, and the partition coefficient and Silhouette index had the highest value.

### Description of identified clusters

As one medical specialty could be part of more than one cluster, each identified cluster was named or labeled based on the specialties that most characterized them. To characterize each cluster, we calculated observed/expected ${((0/E)}_{xy})$ ratios and exclusivity $({EX}_{xy})$ ratios for the medical specialties $x$ within each cluster $y$, following the research of Violán et al. [18]. The ${(0/E)}_{xy}$ ratio is the observed prevalence of medical specialty $x$ in cluster $y$ $({0}_{xy})$ divided by the expected prevalence of medical specialty $x$ in the overall sample $({E}_{x})$. The exclusivity ratio ${EX}_{xy}$ is the sum of the membership degrees of cluster $y$ for patients with the specialty $x$ compared to the total number of patients with the specialty involved (${n}_{x}$). In other words, the exclusivity ratio is the number of individuals with the specialty involved within the cluster divided by the total number of patients with the specialty involved. In line with previous research, we considered a specialty to characterize a cluster when the O/E ratio was ≥2 or the exclusivity value was ≥25% [17, 18, 25]. Further details on the definition of the ratios are provided in Supplementary file 2.

### Membership exploration to identify relevant subgroups

To identify relevant subgroups of the identified clusters, we systematically explored all patients’ membership degrees (‘memberships’) in the clusters of medical specialties. Patients with multimorbidity were the unit of analysis and could belong to different clusters of medical specialties based on their memberships. One medical specialty could be part of more than one cluster and each patient was not assigned to one single cluster but a membership to each cluster.

Memberships were divided into five categories, containing an equal range of memberships (0-20%, 20-40%, 40-60%, 60-80%, and 80-100%). This method was developed and discussed with two data scientists and the research group.

**Fig. 1:**
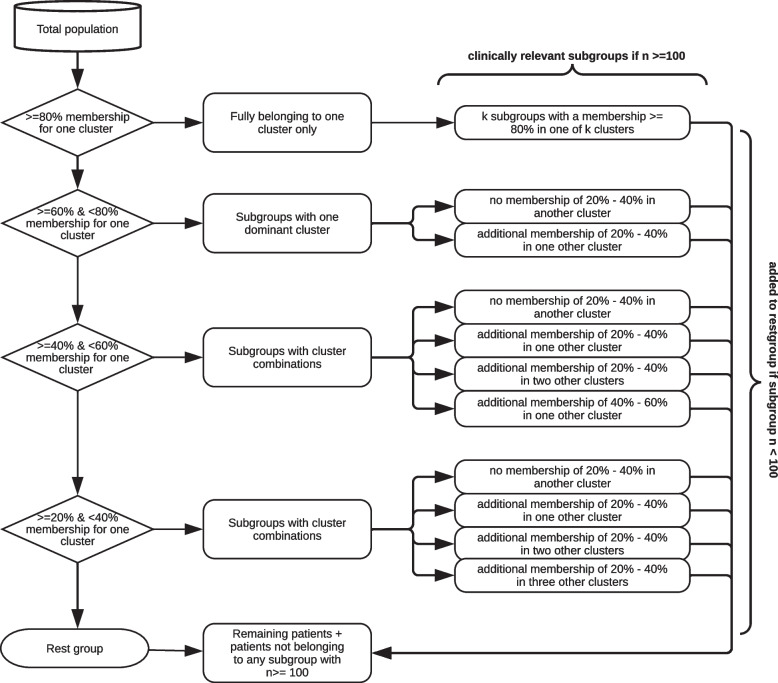
Flowchart of membership degree exploration methodology. Schematic representation of the method that was used for exploration of the population’s membership degrees (‘memberships’) for the clusters, in order to identify relevant subgroups. A membership ≥ 80% was regarded as such a strong membership, that we considered these patients as fully belonging to that cluster. A membership <20% was considered an insignificant membership. A subgroup was considered as potentially clinically relevant if it contained 100 or more patients. The described method of the flowchart is explained in detail in the method section of this paper

We considered patients to fully belong to a cluster if their membership was ≥80%. In contrast, we considered a membership <20% as insignificant. To identify relevant subgroups, we used the method outlined below. We considered a subgroup potentially clinically relevant if it contained 100 or more patients, as we reasoned this number of patients to be a feasible number to consider for a specific hospital intervention. Figure 1 illustrates the following method:

1. We selected all patients who had a membership ≥80% in one of the clusters and considered them to belong to only one cluster fully.
2. Of the remaining patients, we created a subset with all patients with a membership ≥60% and <80% in one of the clusters. We considered these as subgroups with one dominant cluster, having either no or one additional membership between 20-40% in one other cluster.
3. For the remaining patients, we created a subset with all patients with a membership ≥40% and <60% in one of the clusters. These were considered subgroups with cluster combinations where each subgroup could either have no, one, or two additional memberships between 20-40% in one other cluster or one additional membership between 40-60% in one other cluster.
4. We created another subset with all patients with a membership ≥20% and <40% for one of the clusters. These were also cluster combination subgroups, where each subgroup could have no, one, two, or three additional memberships between 20-40% in one other cluster.
5. All remaining patients from this selection process were regarded as the “rest group”, as they did not belong to any subset created in steps one to four. All patients that belonged to a subgroup as outlined before, but where the respective subgroup resulted into a group with less than 100 patients, were also added to the rest group. For the results, we only included the clinically relevant subgroups.

For each subgroup, descriptive statistics and healthcare characteristics were obtained. Frequencies and percentages of involved medical specialties as well as frequencies and percentages of the diseases were calculated per subgroup.

## Results

The dataset contained administrative EHR data from 22,133 patients with multimorbidity with a mean age of 67.9 years (interquartile range (IQR):20.9), and 56.0% were female. Table 1 shows the study population’s baseline characteristics, involvement of medical specialties, and healthcare utilization. Diagnosis prevalence can be found in the table in Supplementary file 3.

**Table 1:** Study population baseline characteristics and involvement of medical specialties (*n=* 22133)

	**Total (*n=*22133)**
**Sex (female)**, n (%)	12396 (56.0)
**Age (in years)**, median (IQR)	67.9 (20.9)
**Age categories (in years)**, n (%)	
18 - 55	5473 (24.7)
56 - 65	4316 (19.5)
66 - 75	6168 (27.9)
75 +	6176 (27.9)
**Number of diagnoses**, n (%)	
2	5286 (23.9)
3	5927 (26.8)
4	4432 (20.0)
5+	6488 (29.3)
**Number of involved medical specialties**, n (%)	
2	7061 (31.9)
3	6982 (31.5)
4	4274 (19.3)
5+	3816 (17.2)
**Medical specialties involved**, n (%)	
Internal medicine	8071 (36.5)
Cardiology	7467 (33.7)
General surgery	6649 (30.0)
Neurology	6266 (28.3)
Pulmonology	5946 (26.9)
Dermatology	5628 (25.4)
Ophthalmology	5295 (23.9)
Otorhinolaryngology	4588 (20.7)
Orthopedic surgery	4423 (20.0)
Urology	3332 (15.1)
Gastroenterology	3083 (13.9)
Rheumatology	2816 (12.7)
Gynecology	2467 (11.2)
Geriatrics	1772 (8.0)
Plastic surgery	1763 (8.0)
Anesthesiology	1455 (6.6)
Dental surgery	1367 (6.2)
Physiatry/ rehabilitation	1130 (5.1)
Psychiatry	262 (1.2)
Neurosurgery	154 (0.7)
Pediatrics	23 (0.1)
Radiology	10 (0.1)
**Number of outpatient visits**, median (IQR)	5.0 (4.0)
**Number of ED visits**, median (IQR)	0.0 (1.0)
**Number of inpatient days**, median (IQR)	0.0 (2.0)

### Identified clusters

To find the optimal cluster solution, we first identified the optimal number of clusters (k) and the fuzziness parameter (m) for the fuzzy c-means algorithm using the four validation indices. We selected the mode of the identified optimal k’s, k=6, as the optimal number of clusters for our analysis with the fuzziness parameter m=1.1. Further details on how the optimal cluster solution was derived are provided in Supplementary file 4 and the figures in Supplementary files 5-7.

**Fig. 2:**
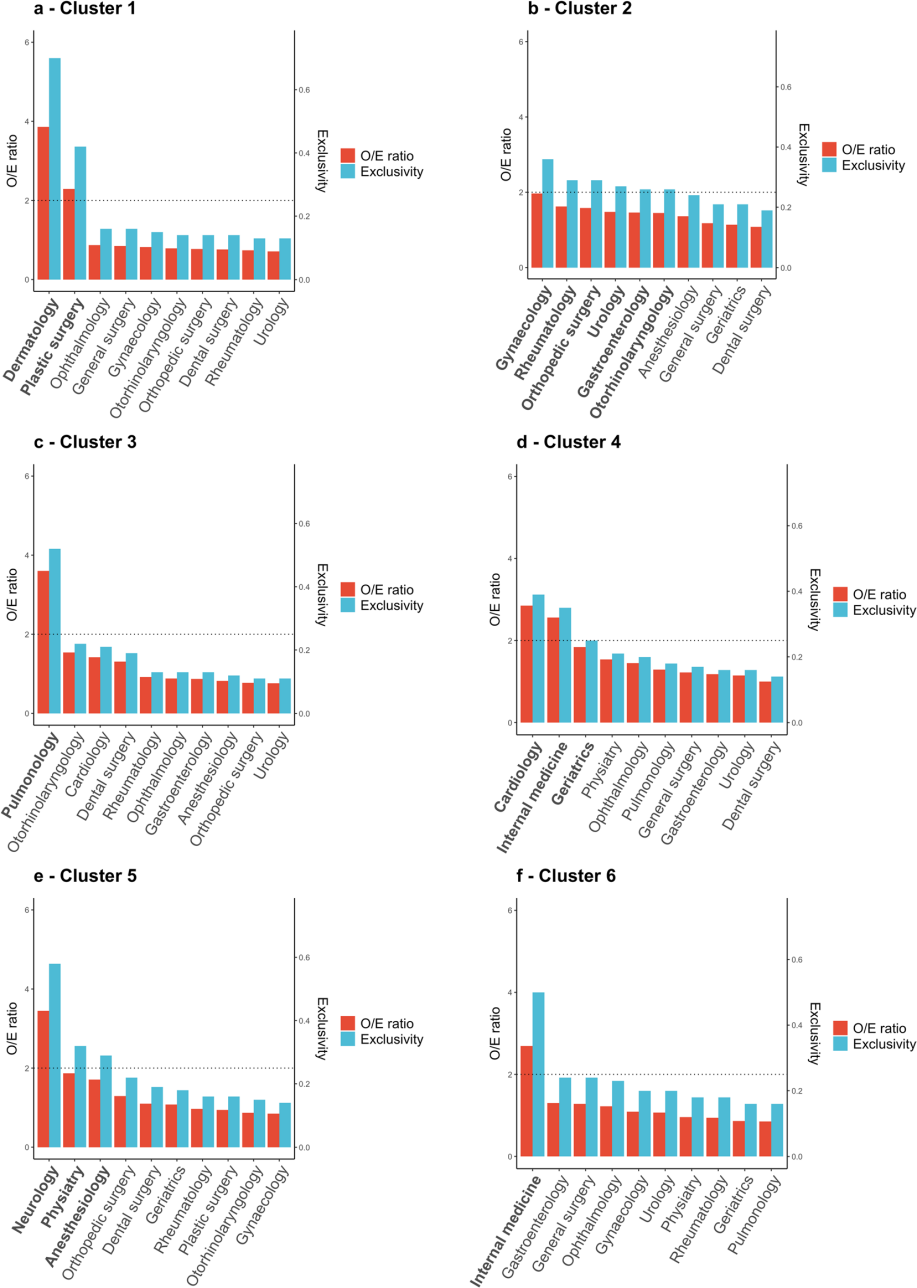
Observed/Expected (O/E) ratio and Exclusivity ratio for 10 most frequent medical specialties in the identified clusters. Medical specialties in bold had an O/E ratio ≥2 or Exclusivity ratio ≥0.25 and therefore characterized the cluster. Dashed lines indicate an O/E ratio ≥2 or Exclusivity ratio ≥0.25. The (O/E)_xy_ ratio is the observed prevalence of medical specialty x in cluster y (O_xy_) divided by the expected prevalence of medical specialty x in the overall sample (E_x_). The exclusivity ratio EX_xy_ is the sum of the membership degrees of cluster y for patients with the specialty x compared to the total number of patients with the specialty involved (n_x_)

We identified six clusters of medical specialties based on the optimal cluster solution. The medical specialties that characterized each of the six clusters are shown in Fig. 2.

To characterize and label each cluster of medical specialties, observed/expected ratios and exclusivity ratios were calculated. With these ratios, we labeled the six clusters of medical specialties based on the specialties that most characterized the clusters:

- Cluster 1: Dermatology and plastic surgery
- Cluster 2: Six specialties (with gynecology, rheumatology, orthopedic surgery, urology, gastroenterology, and otorhinolaryngology)
- Cluster 3: Pulmonology
- Cluster 4: Internal medicine, cardiology, geriatrics
- Cluster 5: Neurology, physiatry (rehabilitation), anesthesiology
- Cluster 6: Internal medicine

### Identified subgroups

**Fig. 3:**
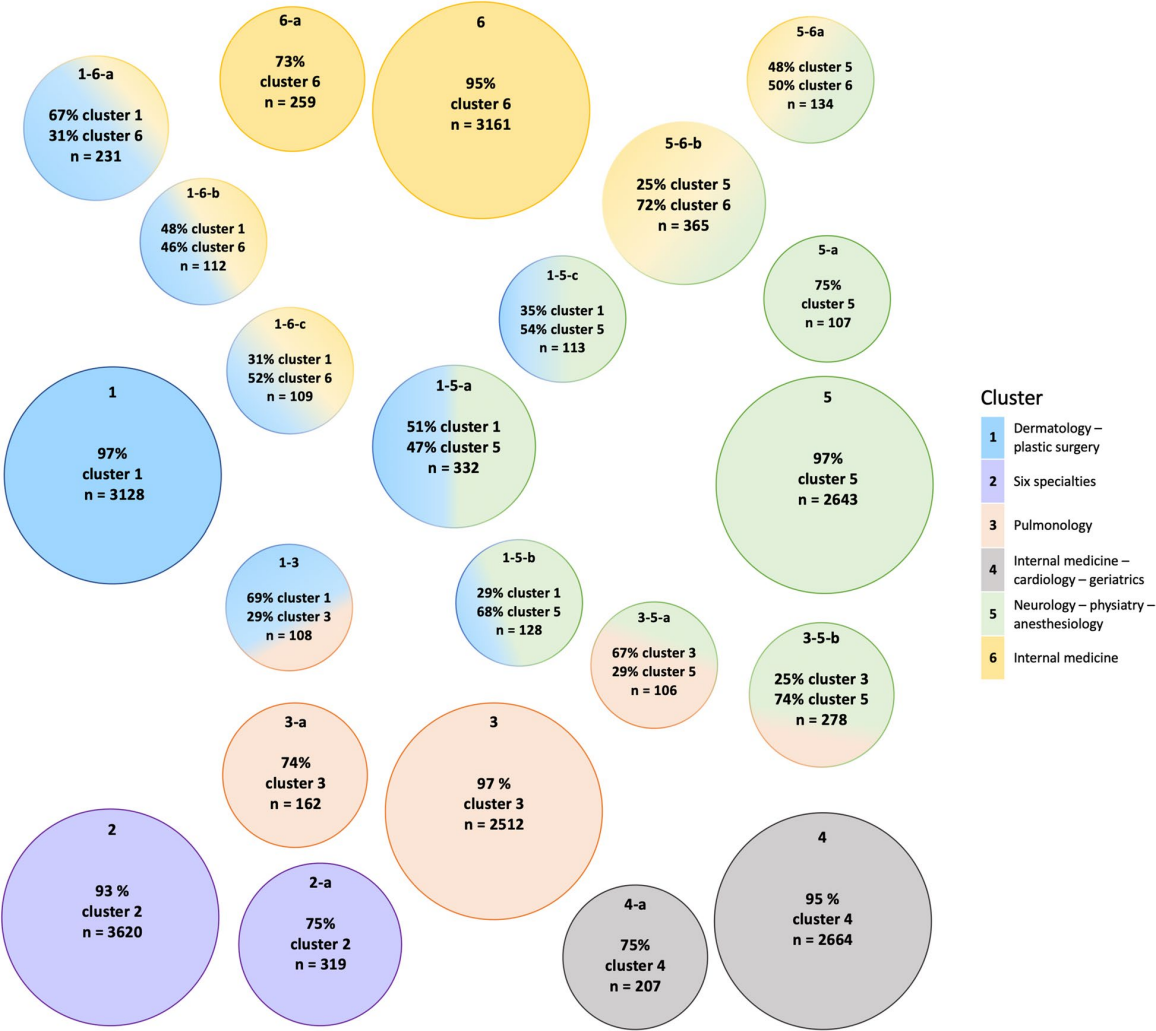
22 identified subgroups of the identified clusters with the mean membership degree in percentages**.** Each circle represents one subgroup. The subgroup’s membership(s) to one or more clusters is/are depicted by the circle’s color(s), each color corresponds with one cluster. In addition, every subgroup is labelled with the subgroup’s name, the mean membership for the cluster(s) and the number of patients in the subgroup. The six larger circles depict the subgroups with a total of 17,728 patients (80.1%) who had a membership of ≥80% (full membership) for one of the six identified clusters. There are five subgroups (2-a to 6-a) with patients with a dominant membership of 60-80% for one of the clusters, but with no clinically relevant membership for another cluster. The other eleven subgroups (1-3, 1-5-a, 1-5-b, 1-5-c, 1-6-a, 1-6-b, 1-6-c, 3-5-a, 3-5-b, 5-6-a and 5-6-b) are combinations of two clusters

We then assigned the total population to subgroups based on the patients’ memberships in each cluster of medical specialties. Figure 3 shows all 22 subgroups with the mean membership for the relevant clusters, illustrating how patients could be members of one or multiple clusters. In our study population, 17,728 patients (80.1%) had a membership of ≥80% for one of the six identified clusters of medical specialties. They were thus in the subgroup of patients with what we defined as a full membership to one cluster only. The patient’s characteristics, frequency, and prevalence of diagnosis groups in each ‘full membership’ subgroup (subgroups 1 to 6) are shown in Table 2.

**Table 2:** Descriptive characteristics for six subgroups of patients with a full membership (≥80%) to one cluster

	**Subgroup 1**	**Subgroup 2**	**Subgroup 3**	**Subgroup 4**	**Subgroup 5**	**Subgroup 6**
**n**	3128	3620	2512	2664	2643	3161
**Membership for cluster**, mean % (SD)	**Cluster 1:** 97 (5)	**Cluster 2:** 93 (5)	**Cluster 3:** 97 (5)	**Cluster 4:** 95 (5)	**Cluster 5:** 97 (4)	**Cluster 6:** 95 (5)
**Sex (female),** n (%)	1767 (56)	2198 (61)	1260 (50)	1237 (46)	1548 (59)	1909 (60)
**Age (in years),** median (IQR)	69 (21.3)	66 (23.2)	67 (17.9)	73 (16.2)	63 (23.5)	65 (19.7)
**Medical specialties involved,** median (IQR)	2 (1)	2 (1)	3 (2)	4 (2)	3 (2)	3 (2)
**Outpatient visits**, median (IQR)	4 (3)	4 (3)	5 (4)	7 (6)	5 (3)	5 (4)
**ED visits**, median (IQR)	0 (0)	0 (0)	0 (1)	1 (2)	0 (0)	0 (1)
**Inpatient days**, median (IQR)	0 (0)	0 (0)	0 (2)	3 (11)	0 (0)	0 (3)
**Diagnoses**, median (IQR)	3 (1)	3 (2)	3 (1)	5 (3)	3 (2)	3 (1)
**Top 5 specialties** (% involved)	1. Dermatology (100) 2. General surgery (24) 3. Plastic surgery (20) 4. Cardiology (17) 5. Ophthalmology (17)	1. General surgery (34) 2. Orthopedic surgery (32) 3. Otorhinolaryngology (32) 4. Cardiology (31) 5. Gynecology (23)	1. Pulmonology (100) 2. Cardiology (51) 3. Otorhinolaryngology (31) 4. Ophthalmology (19) 5. General surgery (18)	1. Internal medicine (100) 2. Cardiology (100) 3. General surgery (36) 4. Pulmonology (34) 5. Ophthalmology (33)	1. Neurology (100) 2. Orthopedic surgery (27) 3. Cardiology (23) 4. General surgery (22) 5. Otorhinolaryngology (18)	1. Internal medicine (100) 2. General surgery (40) 3. Ophthalmology (29) 4. Pulmonology (21) 5. Gastroenterology (18)
**Top 10 diagnoses** (%)	1. Other non-epithelial cancer of skin (51) 2. Other skin disorders (32) 3. Other inflammatory condition of skin (18) 4. Malignant neoplasm without specification of site (17) 5. Residual codes; unclassified (10) 6. Other connective tissue disease (8) 7. Other ear and sense organ disorders (7) 8. Melanomas of skin (7) 9. Cataract (6) 10. Osteoarthritis (5)	1. Residual codes; unclassified (20) 2. Other connective tissue disease (17) 3. Other ear and sense organ disorders (16) 4. Other circulatory disease (11) 5. Spondylosis; intervertebral disc disorders; other back problems (10) 6. Other gastrointestinal disorders (8) 7. Other non-traumatic joint disorders (8) 8. Other female genital disorders (8) 9. Osteoarthritis (7) 10. Other nervous system disorders (7)	1. Other nervous system disorders (28) 2. Asthma (26) 3. Chronic obstructive pulmonary disease and bronchiectasis (26) 4. Other upper respiratory disease (19) 5. Other circulatory disease (15) 6. Residual codes; unclassified (13) 7. Cardiac dysrhythmias (10) 8. Other lower respiratory disease (9) 9. Nonspecific chest pain (8) 10. Other connective tissue disease (8)	1. Residual codes; unclassified (31) 2. Other circulatory disease (25) 3. Congestive heart failure; non-hypertensive (17) 4. Cardiac dysrhythmias (17) 5. Other screening for suspected conditions (not mental disorders or infectious disease) (17) 6. Diabetes mellitus with complications (16) 7. Other aftercare (16) 8. Chronic kidney disease (13) 9. Nonspecific chest pain (13) 10. Other nervous system disorders (12)	1. Other nervous system disorders (32) 2. Spondylosis; intervertebral disc disorders; other back problems (27) 3. Other connective tissue disease (20) 4. Residual codes; unclassified (18) 5. Conditions associated with dizziness or vertigo (11) 6. Headache; including migraine (11) 7. Other ear and sense organ disorders (10) 8. Other non-traumatic joint disorders (9) 9. Other circulatory disease (8) 10. Parkinson's disease (7)	1. Residual codes; unclassified (14) 2. Diabetes mellitus with complications (12) 3. Cancer of breast (10) 4. Other screening for suspected conditions (not mental disorders or infectious disease) (9) 5. Other nervous system disorders (9) 6. Osteoporosis (8) 7. Other connective tissue disease (8) 8. Thyroid disorders (7) 9. Chronic kidney disease (7) 10. Other gastrointestinal disorders (7)

#### Subgroups with full membership to a cluster

Subgroup 1 had a full membership to the dermatology/plastic surgery cluster, with medical specialties mainly involved for skin conditions. In subgroup 2, with full membership to the six specialties cluster, medical specialties were not involved for one specific group of conditions. Instead, they were involved for other, nonspecific problems potentially related to aging, such as other connective tissue diseases, other ear and sense organ disorders, other circulatory diseases, spondylosis intervertebral disc disorders, and back problems. Patients in subgroups 1 and 2 had the lowest number of medical specialties (median:2, IQR:1) and outpatient visits (median:4, IQR:3). In subgroup 3, with full membership to the pulmonology cluster, medical specialties were mainly involved for respiratory diseases, but also for other nervous system disorders and other circulatory diseases. In subgroup 4, with full membership to the internal medicine/cardiology/geriatrics cluster, medical specialists were mainly involved for cardiometabolic diseases. Patients in subgroup 4 had the highest average age (median:73 years, IQR:16.2), highest number of outpatient visits (median:7, IQR:6), and highest number of diagnoses (median:5, IQR:3). It was also the only subgroup with a median greater than zero for ED visits and inpatient days. In subgroup 5, with full membership to the neurology/physiatry (rehabilitation)/anesthesiology cluster, medical specialties were mainly involved for neurological, sensory, and musculoskeletal problems. In subgroup 6, with full membership to the internal medicine cluster, medical specialties were involved for chronic internal medicine conditions, such as thyroid disorders, diabetes mellitus with complications, and breast cancer. The patients in subgroups 5 and 6 had the lowest median age: respectively 63 (IQR:23.5) and 65 (IQR:19.7) years.

#### Subgroups with dominant membership and with no clinically relevant membership to another cluster

We identified five subgroups (2-a to 6-a) with a dominant membership of 60-80% for one of the clusters but with no clinically relevant membership for another cluster. The complete overview of the characteristics of these subgroups with a dominant cluster can be found in Table 3. Most of the average utilization characteristics for these five subgroups (2-a to 6-a) exceeded the average utilization and involvement of medical specialties compared to subgroups 1 to 6 of patients with full cluster membership (Table 2). For example, subgroup 3-a, with a dominant cluster membership to the pulmonology cluster, resembled subgroup 3, but cardiology was more frequently involved, for 75% versus 51%, respectively.

**Table 3:** Descriptive characteristics for five subgroups of patients with a dominant membership (60-80%) to one cluster

	**Subgroup 2-a**	**Subgroup 3-a**	**Subgroup 4-a**	**Subgroup 5-a**	**Subgroup 6-a**
**n**	319	162	207	107	259
**Membership for dominant cluster**, mean % (SD)	**Cluster 2:** 75 (4)	**Cluster 3:** 74 (5)	**Cluster 4:** 75 (4)	**Cluster 5:** 75 (5)	**Cluster 6:** 73 (5)
**Sex (female),** n (%)	164 (51)	86 (53)	104 (50)	54 (50)	152 (59)
**Age (in years),** median (IQR)	74 (20.1)	71 (16.4)	73 (15.0)	70 (20.7)	68 (17.8)
**Medical specialties involved,** median (IQR)	3 (1)	5 (2)	7 (2)	5 (2)	5 (1)
**Outpatient visits**, median (IQR)	6 (3)	9 (5)	12 (7)	8 (5)	9 (6)
**ED visits**, median (IQR)	0 (1)	1 (1)	1 (2)	0 (1)	1 (2)
**Inpatient days**, median (IQR)	0 (2)	0 (6)	9 (21)	0 (7)	3 (11)
**Diagnoses**, median (IQR)	4 (2)	6 (2)	9 (4)	6 (3)	6 (2)
**Top 5 specialties** (% involved)	1. Cardiology (65) 2. Ophthalmology (65) 3. General surgery (32) 4. Urology (24) 5. Physiatry (24)	1. Pulmonology (100) 2. Cardiology (75) 3. General surgery (55) 4. Neurology (41) 5. Ophthalmology (33)	1. Cardiology (100) 2. Internal medicine (100) 3. Neurology (51) 4. Orthopedic surgery (51) 5. Pulmonology (43)	1. Neurology (100) 2. Cardiology (56) 3. General surgery (42) 4. Ophthalmology (38) 5. Pulmonology (34)	1. Internal medicine (100) 2. Pulmonology (68) 3. Neurology (55) 4. General surgery (52) 5. Ophthalmology (45)
**Top 10 diagnoses** (%)	1. Residual codes; unclassified (36) 2. Cataract (24) 3. Other connective tissue disease (19) 4. Other eye disorders (18) 5. Other circulatory disease (16) 6. Spondylosis; intervertebral disc disorders; other back problems (13) 7. Other nervous system disorders (13) 8. Cardiac dysrhythmias (12) 9. Retinal detachments; defects; vascular occlusion; and retinopathy (11) 10. Other non-traumatic joint disorders (11)	1. Other nervous system disorders (30) 2. Residual codes; unclassified (27) 3. Asthma (23) 4. Chronic obstructive pulmonary disease and bronchiectasis (23) 5. Other circulatory disease (22) 6. Other screening for suspected conditions (not mental disorders or infectious disease) (15) 7. Other non-epithelial cancer of skin (15) 8. Cardiac dysrhythmias (15) 9. Spondylosis; intervertebral disc disorders; other back problems (14) 10. Other skin disorders (13)	1. Residual codes; unclassified (43) 2. Other screening for suspected conditions (not mental disorders or infectious disease) (27) 3. Other circulatory disease (26) 4. Cardiac dysrhythmias (26) 5. Spondylosis; intervertebral disc disorders; other back problems (25) 6. Other nervous system disorders (24) 7. Other aftercare (18) 8. Other upper respiratory disease (15) 9. Other connective tissue disease (15) 10. Diabetes mellitus with complications (14)	1. Other nervous system disorders (35) 2. Spondylosis; intervertebral disc disorders; other back problems (33) 3. Residual codes; unclassified (30) 4. Other connective tissue disease (17) 5. Other eye disorders (15) 6. Other circulatory disease (14) 7. Other screening for suspected conditions (not mental disorders or infectious disease) (12) 8. Cardiac dysrhythmias (11) 9. Acute cerebrovascular disease (10) 10. Delirium dementia and amnestic and other cognitive disorders (10)	1. Residual codes; unclassified (29) 2. Other nervous system disorders (27) 3. Spondylosis; intervertebral disc disorders; other back problems (17) 4. Other screening for suspected conditions (not mental disorders or infectious disease) (17) 5. Chronic obstructive pulmonary disease and bronchiectasis (14) 6. Osteoarthritis (14) 7. Asthma (14) 8. Pneumonia (except that caused by tuberculosis or sexually transmitted disease) (13) 9. Diabetes mellitus with complications (13) 10. Other eye disorders (12)

#### Subgroups with membership to two clusters

The other eleven subgroups were combinations of two clusters, for which the complete overview of the characteristics can be found in Tables 4. and 5.. The median number of specialties ranged from 3 to 5 and the median number of outpatient visits from 4 to 9. For all these subgroups, the median inpatient days were zero (IQR ranging from 0-8).

**Table 4:** Descriptive characteristics for six of the eleven subgroups of patients with combinations of clusters

	**Combination cluster 1 & 5**			**Combination cluster 1 & 6**		
	**Subgroup 1-5-a**	**Subgroup 1-5-b **	**Subgroup 1-5-c**	**Subgroup 1-6-a**	**Subgroup 1-6-b**	**Subgroup 1-6-c**
**n**	332	128	113	231	112	109
**Memberships for clusters**, mean % (SD)	**Cluster 1: **51 (5) **Cluster 5: **47 (4)	**Cluster 1:** 29 (5) **Cluster 5:** 68 (5)	**Cluster 1: **35 (5) **Cluster 5: **54 (6)	**Cluster 1**: 67 (6) **Cluster 6**: 31 (6)	**Cluster 1: **48 (4) **Cluster 6: **46 (4)	**Cluster 1: **31 (5) **Cluster 6: **52 (5)
**Sex (female)**, n (%)	179 (54)	79 (62)	45 (40)	133 (58)	67 (60)	65 (60)
**Age (in years)**, median (IQR)	69 (22.3)	71 (21.7)	71 (19.2)	69 (17.9)	69 (15.9)	70 (16.6)
**Medical specialties involved, **median (IQR)	3 (2)	4 (2)	5 (3)	3 (1)	4 (1)	5 (1)
**Outpatient visits**, median (IQR)	5 (4)	6 (3)	7 (6)	6 (4)	7 (5)	9 (6)
**ED visits**, median (IQR)	0 (0)	0 (0)	0 (1)	0 (0)	0 (1)	1 (1)
**Inpatient days**, median (IQR)	0 (0)	0 (0)	0 (3)	0 (0)	0 (6)	0 (8)
**Diagnoses**, median (IQR)	3 (2)	4 (1)	5 (3)	4 (1)	4 (1)	6 (2)
**Top 5 specialties **(% involved)	1. Dermatology (100) 2. Neurology (100) 3. Ophthalmology (26) 4. Surgeon (17) 5. Otorhinolaryngology (17)	1. Dermatology (100) 2. Neurology (100) 3. Orthopedic surgery (76) 4. Anesthesiology (20) 5. Physiatry (18)	1. Dermatology (100) 2. Neurology (100) 3. Cardiology (66) 4. Ophthalmology (31) 5. General surgery (26)	1. Dermatology (100) 2. Internal medicine (100) 3. Ophthalmology (35) 4. General surgery (30) 5. Urology (16)	1. Dermatology (100) 2. Internal medicine (100) 3. General surgery (48) 4. Pulmonology (27) 5. Ophthalmology (22)	1. Dermatology (100) 2. Internal medicine (100) 3. Ophthalmology (58) 4. General surgery (42) 5. Neurology (34)
**Top 10 diagnoses** (%)	1. Other skin disorders (39) 2. Other non-epithelial cancer of skin (38) 3. Other nervous system disorders (26) 4. Spondylosis; intervertebral disc disorders; other back problems (19) 5. Other inflammatory condition of skin (18) 6. Conditions associated with dizziness or vertigo (11) 7. Headache; including migraine (10) 8. Parkinson's disease (9) 9. Other ear and sense organ disorders (8) 10. Cataract (8)	1. Other skin disorders (41) 2. Spondylosis; intervertebral disc disorders; other back problems (38) 3. Other non-epithelial cancer of skin (32) 4. Other nervous system disorders (30) 5. Osteoarthritis (30) 6. Residual codes; unclassified (24) 7. Other connective tissue disease (23) 8. Other inflammatory condition of skin (20) 9. Conditions associated with dizziness or vertigo (9) 10. Headache; including migraine (7)	1. Other non-epithelial cancer of skin (39) 2. Other nervous system disorders (32) 3. Other skin disorders (31) 4. Other inflammatory condition of skin (26) 5. Spondylosis; intervertebral disc disorders; other back problems (21) 6. Residual codes; unclassified (19) 7. Nonspecific chest pain (16) 8. Other eye disorders (13) 9. Other screening for suspected conditions (not mental disorders or infectious disease) (12) 10. Other connective tissue disease (12)	1. Other skin disorders (35) 2. Other non-epithelial cancer of skin (34) 3. Other inflammatory condition of skin (26) 4. Cancer of breast (12) 5. Residual codes; unclassified (11) 6. Other screening for suspected conditions (not mental disorders or infectious disease) (11) 7. Osteoporosis (10) 8. Cataract (9) 9. Diabetes mellitus with complications (9) 10. Chronic kidney disease (8)	1. Other skin disorders (27) 2. Other non-epithelial cancer of skin (27) 3. Other inflammatory condition of skin (21) 4. Residual codes; unclassified (16) 5. Other and unspecified benign neoplasm (13) 6. Osteoporosis (12) 7. Phlebitis; thrombophlebitis and thromboembolism (10) 8. Allergic reactions (9) 9. Asthma (9) 10. Cancer of breast (8)	1. Other skin disorders (32) 2. Other inflammatory condition of skin (28) 3. Other non-epithelial cancer of skin (27) 4. Residual codes; unclassified (22) 5. Cataract (18) 6. Diabetes mellitus with complications (17) 7. Other screening for suspected conditions (not mental disorders or infectious disease) (16) 8. Other eye disorders (15) 9. Other nervous system disorders (11) 10. Other and unspecified benign neoplasm (11)

**Table 5:** Descriptive characteristics for five of the eleven subgroups of patients with combinations of clusters

	**Combination cluster 3 & 5**		**Combination cluster 5 & 6**		**Combination cluster 1 & 3**
	**Subgroup 3-5-a**	**Subgroup 3-5-b **	**Subgroup 5-6-a**	**Subgroup 5-6-b**	**Subgroup 1-3**
**n**	106	278	134	365	108
**Memberships for clusters**, mean % (SD)	**Cluster 3:** 67 (3) **Cluster 5:** 29 (5)	**Cluster 3: **25 (5) **Cluster 5: **74 (6)	**Cluster 5: **48 (6) **Cluster 6:** 50 (6)	**Cluster 5: **25 (4) **Cluster 6: **72 (6)	**Cluster 1:** 69 (5) **Cluster 3:** 29 (4)
**Sex (female)**, n (%)	43 (41)	155 (56)	89 (66)	225 (62)	66 (61)
**Age (in years)**, median (IQR)	70 (18.1)	61 (21.4)	62 (23.5)	62 (25.1)	68 (17.3)
**Medical specialties involved, **median (IQR)	4 (2)	3 (1)	3 (1)	3 (2)	3 (1)
**Outpatient visits**, median (IQR)	8 (5)	4 (4)	6 (4)	5 (5)	6 (4)
**ED visits**, median (IQR)	0 (1)	0 (1)	0 (1)	0 (1)	0 (0)
**Inpatient days**, median (IQR)	0 (5)	0 (0)	0 (2)	0 (3)	0 (0)
**Diagnoses**, median (IQR)	5 (3)	3 (2)	4 (2)	3 (3)	4 (1)
**Top 5 specialties **(% involved)	1. Neurology (100) 2. Pulmonology (100) 3. Cardiology (54) 4. Otorhinolaryngology (52) 5. Ophthalmology (19)	1. Neurology (100) 2. Pulmonology (100) 3. General surgery (21) 4. Ophthalmology (14) 5. Rheumatology (10)	1. Internal medicine (100) 2. Neurology (100) 3. Orthopedic surgery (43) 4. Anesthesiology (31) 5. Otorhinolaryngology (28)	1. Internal medicine (100) 2. Neurology (100) 3. Ophthalmology (18) 4. Urology (17) 5. Orthopedic surgery (12)	1. Dermatology (100) 2. Pulmonology (100) 3. Ophthalmology (39) 4. General surgery (25) 5. Orthopedic surgery (19)
**Top 10 diagnoses** (%)	1. Other nervous system disorders (47) 2. Chronic obstructive pulmonary disease and bronchiectasis (30) 3. Other upper respiratory disease (25) 4. Residual codes; unclassified (22) 5. Asthma (17) 6. Other circulatory disease (14) 7. Spondylosis; intervertebral disc disorders; other back problems (13) 8. Cardiac dysrhythmias (12) 9. Other aftercare (12) 10. Other connective tissue disease (10)	1. Other nervous system disorders (38) 2. Asthma (28) 3. Spondylosis; intervertebral disc disorders; other back problems (19) 4. Chronic obstructive pulmonary disease and bronchiectasis (17) 5. Conditions associated with dizziness or vertigo (15) 6. Cancer of bronchus; lung (15) 7. Headache; including migraine (13) 8. Other upper respiratory disease (12) 9. Pneumonia (except that caused by tuberculosis or sexually transmitted disease) (9) 10. Other connective tissue disease (7)	1. Spondylosis; intervertebral disc disorders; other back problems (44) 2. Other nervous system disorders (26) 3. Other connective tissue disease (18) 4. Residual codes; unclassified (16) 5. Headache; including migraine (11) 6. Other ear and sense organ disorders (10) 7. Osteoporosis (10) 8. Osteoarthritis (9) 9. Essential hypertension (9) 10. Other screening for suspected conditions (not mental disorders or infectious disease) (8)	1. Other nervous system disorders (34) 2. Spondylosis; intervertebral disc disorders; other back problems (16) 3. Residual codes; unclassified (14) 4. Essential hypertension (9) 5. Headache; including migraine (9) 6. Other screening for suspected conditions (not mental disorders or infectious disease) (8) 7. Diabetes mellitus with complications (8) 8. Osteoporosis (7) 9. Malaise and fatigue (7) 10. Other connective tissue disease (7)	1. Other skin disorders (36) 2. Other non-epithelial cancer of skin (34) 3. Asthma (28) 4. Chronic obstructive pulmonary disease and bronchiectasis (24) 5. Other nervous system disorders (19) 6. Other inflammatory condition of skin (18) 7. Cataract (13) 8. Other connective tissue disease (11) 9. Other and unspecified benign neoplasm (11) 10. Other eye disorders (8)

A combination with cluster 1 (dermatology and plastic surgery) was present in seven of the eleven subgroups (1-3, 1-5-a, 1-5-b, 1-5-c, 1-6-a, 1-6-b, and 1-6-c), although no dominant subgroup for cluster 1 was identified. Three subgroups were a combination of clusters 1 and 5 (neurology, physiatry (rehabilitation), anesthesiology), and all patients in these subgroups had seen a neurologist and a dermatologist (subgroups 1-5-a, 1-5-b, and 1-5-c). In all three subgroups, similar diagnosis groups were present. In subgroup 1-5-b, 76% of the patients had also seen an orthopedic surgeon, with osteoarthritis in the top 10 diagnoses. Most patients in this subgroup were female (62%). In subgroup 1-5-c, 66% had also seen a cardiologist, with nonspecific chest pain in the top 10 diagnoses, and the majority was male (60%).

Three subgroups were a combination of cluster 1 with cluster 6 (internal medicine). In these subgroups (1-6-a, 1-6-b, and 1-6-c), the dermatologist and internist were involved for all patients. Other non-epithelial cancer, inflammatory conditions, and other disorders of the skin were among the top 10 diagnoses. Subgroup 1-6-c had the highest median number of diagnoses: 6 (IQR:2) compared to 4 (IQR:1) in the other two subgroups (1-6-a and 1-6-b).

There was only one subgroup with clusters 1 and 3 (pulmonology). Pulmonology and dermatology were involved for all patients in this subgroup 1-3. The top four diagnoses were other skin disorders, other non-epithelial cancer of the skin, asthma, chronic obstructive pulmonary disease, and bronchiectasis.

A combination with cluster 5 (neurology, physiatry (rehabilitation), anesthesiology) was present in the remaining four subgroups (3-5-a, 3-5-b, 5-6-a, and 5-6-b). The combination with cluster 3 (pulmonology) was found in two subgroups, where the pulmonologist and neurologist were involved for all patients (subgroups 3-5-a and 3-5-b). Other nervous system disorders, chronic obstructive pulmonary disease, and asthma were in the top 10 diagnoses in both subgroups. However, in subgroup 3-5-a, the cardiologist and otorhinolaryngologist were also involved for around half of the patients. Subgroup 3-5-a had a median age of 70 years (IQR:18.1), 25% had the diagnosis of other upper respiratory disease, and 12% had the diagnosis of cardiac dysrhythmias. The patients in the subgroup 3-5-b had a median age of 61 years (IQR:21.4). Subgroup 3-5-b was the only subgroup with pneumonia, cancer of bronchus or lung, headache, and conditions associated with dizziness in the top 10 diagnoses.

Finally, the combination of clusters 5 (neurology, physiatry (rehabilitation), anesthesiology) and 6 (internal medicine) was found in two subgroups (5-6-a and 5-6-b). In both subgroups, the internist and the neurologist were involved for all patients. Subgroup 5-6-b was the largest of all eleven subgroups, with 365 patients. This subgroup had diabetes mellitus with complications, malaise, and fatigue in the top 10 diagnoses. Headache (including migraine), essential hypertension, and osteoporosis were present in the top 10 diagnoses in both subgroups.

The remaining seven percent of the study population (*n=*1,538) did not belong to any identified subgroups and are therefore not further specified.

## Discussion

This study used fuzzy c-means cluster analysis to explore the complexity of multimorbidity and the simultaneous involvement of multiple medical specialties in the hospital. We found six clusters of medical specialties and identified 22 subgroups with a membership exploration method. Most patients (80%) belonged to a subgroup with full membership (≥80%) to one cluster of medical specialties. The subgroups with a full or dominant membership to the clusters resembled previously identified disease clusters, such as COPD and asthma (subgroup 3 and 3a); cardiometabolic disorders (subgroup 4 and 4a) and osteoporosis, back pain, musculoskeletal disorders, and soft tissue disorders (subgroup 5) [7].

Approximately 15% of the patients belonged to subgroups with a dominant cluster membership (but <80%) or a combination of cluster memberships. Further examination showed that the prevalence of specific diagnoses, patient characteristics, and healthcare utilization can differ between subgroups. The subgroups’ differences in characteristics provide clues about potential target populations who might benefit most from (more) multidisciplinary collaboration. Medical specialists can use the subgroups to discuss whether they can explain their simultaneous involvement and to explore whether the current hospital care is sufficiently coordinated. Subgroups of interest are those with many patients and involved medical specialties and with higher healthcare utilization. Multidisciplinary collaboration for these subgroups could prevent or reduce adverse treatment interactions and fragmented care.

To illustrate how the exploration of subgroups might lead to new ideas for coordination and the organization of multidisciplinary collaboration, we present two examples. In subgroups 3 and 3a, the cardiologist and pulmonologist were involved for almost 1500 patients. The prevalent diagnoses of asthma and chronic obstructive pulmonary disease are not directly related to heart diseases. However, they share risk factors, and diseases and treatments can interact and present with the same symptoms [26, 27]. Consequently, more or another type of collaboration between medical specialties might be necessary to reach timely diagnoses and coordinated treatment plans, as shown by Rietbroek et al. [28] for the dyspnea clinic.

Another example can be found in subgroups 4 and 4a, with patients with memberships in the internal medicine, cardiology, and geriatrics cluster, who were treated for cardiometabolic diseases. These subgroups could open the discussion about formalizing collaboration in a cardiometabolic outpatient clinic as proposed by Reiter-Brennan et al. [29]. Moreover, this discussion is even more relevant if these subgroups, as we identified, use more healthcare compared to other subgroups. Future research should focus on whether some healthcare utilization could be reduced by improving collaboration.

Another clue for the potential benefit of enhanced multidisciplinary collaboration can be the presence of unrecognized interactions of diseases and treatments. Involved medical specialties could investigate whether they can identify underlying causes and reasons for their shared involvement, including the risk of interactions. Skin conditions are related to aging but can also be caused by medication or treatments [30]. Patients with full membership for cluster 1 (dermatology and general surgery) were slightly older but used little hospital care. In contrast, patients in subgroups with a combination of cluster 1 and another cluster seem to use more healthcare. By discussing whether the diseases and treatments in these latter subgroups interact, medical specialists can investigate whether (more) multidisciplinary collaboration might help improve coordination and the quality of their healthcare delivery.

Finally, the subgroups can help to discuss and coordinate the approach to problems in domains other than the medical domains. For example, multimorbidity is associated with functional decline [5]. The top ten diagnoses in the subgroups with combinations of cluster 5 (neurology, physiatry (rehabilitation), anesthesiology) and cluster 6 (internal medicine) contain multiple diagnoses that could impact daily functioning. The neurologist and internist, both involved for all patients in these subgroups, could discuss how they incorporate the evaluation of and support for functional decline among their patients.

One strength of this study is that the fuzzy c-means algorithm is less susceptible to outliers, the choice of distance measure, and the inclusion of irrelevant variables compared to hierarchical cluster analysis [31]. Furthermore, cluster analysis is more suitable than exploratory factor or latent class analysis when health conditions within clusters are not assumed to be causally related and the data is not in a continuous format [7, 9]. Another strength of this study is the inclusion of real-world data from an entire hospital population with multimorbidity. At the same time, this also brings some limitations.

One limitation of examining the clusters’ and subgroups’ characteristics is the generic description of several diagnosis groups, combined with the overrepresentation of ‘other’ diagnosis groups. Detailed information is missing, partly due to the classification systems used. The quality and details of the data also depend on how data were registered. An alternative explanation for the overrepresentation of ‘other’ diagnosis groups is the difficulty that care professionals may experience in attributing a patient’s complaint to a specific diagnosis because of the complexity and atypical disease presentation of multimorbidity [32].

Another limitation is that the data does not distinguish between the involvement of medical specialists in outpatient versus inpatient clinical care. A sub-analysis for outpatient clinical care might result in other clusters or subgroups, which could be relevant if a hospital only wants to focus on improving multidisciplinary collaboration at the outpatient clinic. Additionally, data was collected from a single hospital and did not provide information on (missing) collaboration among professionals across different healthcare organizations.

Furthermore, the generalizability of this research to other healthcare organizations needs further exploration. We recommend applying our proposed methodology across multiple healthcare organizations to explore and discuss regional or national multidisciplinary collaboration for patients with multimorbidity. Although this study was performed in a clinical department with medical specialists involved in the innovation of hospital care, the clinical applicability of our explorative methodology should be discussed with relevant stakeholders and involved healthcare providers. Further, the number and type of clusters can differ across hospitals because of differences in care delivery per hospital or the employed clustering method. Future research should explore whether our results are replicable with other clustering methods. In addition, our explorative methodology should be applied across different hospitals to study similarities and differences in cluster and subgroup results. Nevertheless, our proposed explorative methodology can be used to start unraveling the complexity of multimorbidity within and across hospitals to improve multidisciplinary collaboration.

Moreover, different cut-off values for the O/E ratio, exclusivity, and number of membership groups for membership exploration might lead to different results. Based on previous literature, we chose an O/E ratio ≥2 and an exclusivity value of ≥25% [17, 18, 25]. For most clusters, these cut-off values led to a low number of medical specialties characterizing a cluster, enabling a relatively manageable description of clusters.

Finally, changing the number of membership categories used for membership exploration, for example into ten (0-10%, 10-20%, etc.) or four (0-25%, 25-50%, etc.) membership categories instead of five membership categories (0-20%, 20-40%, etc.), changes the possibility of multiple combinations of clusters. More categories might offer more detailed information but could also complicate interpretation and result in small and clinically irrelevant subgroups. Fewer categories might simplify the results but allow for less overlap or combinations of clusters.

## Conclusions

To the best of our knowledge, this is the first study that used fuzzy c-means to cluster medical specialties involved in the care for patients with multimorbidity and that explored the distribution of membership degrees to identify subgroups of clusters. Every hospital can use fuzzy c-means cluster analysis and clinical data from their EHR to identify these clusters and subgroups. Clusters and subgroups differed regarding the involved medical specialties, diagnoses, patient characteristics, and healthcare utilization. Our strategy can help hospitals and medical specialists to discuss simultaneous involvement and identify potential target populations with multimorbidity that might benefit from improved multidisciplinary collaboration.

### Supplementary Information

**Additional file 1: Supplementary file 1.** Table of the 233 diagnosis groups used in this study from the Dutch Hospital Data-Clinical Classification Software (DHD-CCS). **Supplementary file 2.** Definition of observed/expected ratios and exclusivity ratios. **Supplementary file 3.** Diagnoses with a prevalence greater than 2% in the study population (n = 22133). **Supplementary file 4.** Optimal parameters for fuzzy c-means. **Supplementary file 5.** Validation indices for m= 1.1, 1.2, 1.3, 1.4, 1.5. **Supplementary file 6.** Validation indices for m= 1.1, 1.2, 1.5 (for Xie-Beni: only 1.1 & 1.2). **Supplementary file 7.** Validation indices for m= 1.1.

## Data Availability

The data that support the findings of this study are available from Gelre hospitals Apeldoorn but restrictions apply to the availability of these data, which were used under license for the current study, and so are not publicly available. Data are however available from the authors (via the corresponding author, Liann Weil: l.i.weil@umcg.nl) upon reasonable request and with permission of Gelre hospitals Apeldoorn.

## Abbreviations

DHD-CCS: Dutch Hospital Data – Clinical Classification Software
DTC: Diagnosis Treatment Combinations
ED: Emergency Department
EX: Exclusivity
IQR: Interquartile Range
O/E: Observed/Expected
