# Supplementary material for: Clusters of medical specialties around patients with multimorbidity – employing fuzzy c-means clustering to explore multidisciplinary collaboration
Source: BMC Health Serv Res. 2023 Sep 9;23:975. doi: 10.1186/s12913-023-09961-z (PMC10492354; doi:10.1186/s12913-023-09961-z)

SUPPLEMENTARY FILES

Clusters of medical specialties around patients with multimorbidity – employing fuzzy c-means clustering to explore multidisciplinary collaboration

Content

Supplementary file 1 – Table of the 233 diagnosis groups used in this study from the Dutch Hospital Data-Clinical Classification Software (DHD-CCS) 2

Supplementary file 2 – Definition of observed/expected ratios and exclusivity ratios 6

Supplementary file 3 – Diagnoses with a prevalence greater than 2% in the study population (n = 22133) 7

Supplementary file 4 - Optimal parameters for fuzzy c-means 8

Supplementary file 5 - Validation indices for m= 1.1, 1.2, 1.3, 1.4, 1.5 9

Supplementary file 6 - Validation indices for m= 1.1, 1.2, 1.5 (for Xie-Beni: only 1.1 & 1.2) 10

Supplementary file 7 - Validation indices for m= 1.1 11

Supplementary file 1 – Table of the 233 diagnosis groups used in this study from the Dutch Hospital Data-Clinical Classification Software (DHD-CCS)

	Diagnosis group	Diagnosis type
1	Abdominal hernia	Elective
2	Abdominal pain	Chronic
3	Acquired foot deformities	Chronic
4	Acute and chronic tonsillitis	Chronic
5	Acute and unspecified renal failure	Acute
6	Acute bronchitis	Acute
7	Acute cerebrovascular disease	Acute
8	Acute myocardial infarction	Acute
9	Adjustment disorders	Chronic
10	Administrative/social admission	Other
11	Alcohol-related disorders	Other
12	Allergic reactions	Chronic
13	Anal and rectal conditions	Elective
14	Anxiety disorders	Chronic
15	Aortic and peripheral arterial embolism or thrombosis	Acute
16	Aortic; peripheral; and visceral artery aneurysms	Acute
17	Appendicitis and other appendiceal conditions	Acute
18	Asthma	Chronic
19	Bacterial infection; unspecified site	Acute
20	Benign neoplasm of uterus	Elective
21	Biliary tract disease	Elective
22	Birth trauma	Other
23	Blindness and vision defects	Chronic
24	Burns	Acute
25	Calculus of urinary tract	Elective
26	Cancer of bladder	Oncologic
27	Cancer of bone and connective tissue	Oncologic
28	Cancer of brain and nervous system	Oncologic
29	Cancer of breast	Oncologic
30	Cancer of bronchus; lung	Oncologic
31	Cancer of cervix	Oncologic
32	Cancer of colon	Oncologic
33	Cancer of esophagus	Oncologic
34	Cancer of head and neck	Oncologic
35	Cancer of kidney and renal pelvis	Oncologic
36	Cancer of liver and intrahepatic bile duct	Oncologic
37	Cancer of other female genital organs	Oncologic
38	Cancer of other GI organs; peritoneum	Oncologic
39	Cancer of other male genital organs	Oncologic
40	Cancer of other urinary organs	Oncologic
41	Cancer of ovary	Oncologic
42	Cancer of pancreas	Oncologic
43	Cancer of prostate	Oncologic
44	Cancer of rectum and anus	Oncologic
45	Cancer of stomach	Oncologic
46	Cancer of testis	Oncologic
47	Cancer of thyroid	Oncologic
48	Cancer of uterus	Oncologic
49	Cancer; other and unspecified primary	Oncologic
50	Cancer; other respiratory and intrathoracic	Oncologic
51	Cardiac and circulatory congenital anomalies	Chronic
52	Cardiac arrest and ventricular fibrillation	Acute
53	Cardiac dysrhythmias	Acute
54	Cataract	Elective
55	Chronic kidney disease	Chronic
56	Chronic obstructive pulmonary disease and bronchiectasis	Chronic
57	Chronic ulcer of skin	Chronic
58	Coagulation and hemorrhagic disorders	Chronic
59	Coma; stupor; and brain damage	Acute
60	Complication of device; implant or graft	Other
61	Complications of surgical procedures or medical care	Acute
62	Conditions associated with dizziness or vertigo	Chronic
63	Conduction disorders	Chronic
64	Congestive heart failure; non-hypertensive	Chronic
65	Contraceptive and procreative management	Elective
66	Coronary atherosclerosis and other heart disease	Chronic

Supplementary file 1 – continued		
67	Crushing injury or internal injury	Acute
68	Cystic fibrosis	Chronic
69	Deficiency and other anemia	Chronic
70	Delirium dementia and amnestic and other cognitive disorders	Chronic
71	Diabetes mellitus with complications	Chronic
72	Diabetes mellitus without complication	Chronic
73	Diabetes or abnormal glucose tolerance complicating pregnancy; childbirth; or the puerperium	Chronic
74	Digestive congenital anomalies	Acute
75	Diseases of mouth; excluding dental	Elective
76	Diseases of white blood cells	Chronic
77	Disorders of lipid metabolism	Chronic
78	Disorders of teeth and jaw	Other
79	Disorders usually diagnosed in infancy childhood or adolescence	Chronic
80	Diverticulosis and diverticulitis	Chronic
81	Ectopic pregnancy	Other
82	Encephalitis (except that caused by tuberculosis or sexually transmitted disease)	Acute
83	Endometriosis	Chronic
84	Epilepsy; convulsions	Chronic
85	Esophageal disorders	Chronic
86	Essential hypertension	Chronic
87	Female infertility	Elective
88	Fever of unknown origin	Acute
89	Fluid and electrolyte disorders	Other
90	Fracture of lower limb	Acute
91	Fracture of neck of femur (hip)	Acute
92	Fracture of upper limb	Acute
93	Gastritis and duodenitis	Chronic
94	Gastroduodenal ulcer (except hemorrhage)	Elective
95	Gastrointestinal hemorrhage	Chronic
96	Genitourinary congenital anomalies	Chronic
97	Genitourinary symptoms and ill-defined conditions	Chronic
98	Glaucoma	Elective
99	Gout and other crystal arthropathies	Chronic
100	Headache; including migraine	Chronic
101	Heart valve disorders	Elective
102	Hemolytic jaundice and perinatal jaundice	Acute
103	Hemorrhoids	Elective
104	Hepatitis	Chronic
105	HIV infection	Chronic
106	Hodgkin`s disease	Oncologic
107	Hyperplasia of prostate	Chronic
108	Hypertension complicating pregnancy; childbirth and the puerperium	Other
109	Immunity disorders	Chronic
110	Immunizations and screening for infectious disease	Acute
111	Infective arthritis and osteomyelitis (except that caused by tuberculosis or sexually transmitted disease)	Chronic
112	Inflammation; infection of eye (except that caused by tuberculosis or sexually transmitted disease)	Acute
113	Inflammatory conditions of male genital organs	Acute
114	Inflammatory diseases of female pelvic organs	Acute
115	Intestinal infection	Acute
116	Intestinal obstruction without hernia	Acute
117	Intracranial injury	Acute
118	Joint disorders and dislocations; trauma-related	Acute
119	Late effects of cerebrovascular disease	Other
120	Leukemias	Oncologic
121	Liver disease, alcohol-related	Chronic
122	Lung disease due to external agents	Chronic
123	Lymphadenitis	Chronic
124	Malaise and fatigue	Chronic
125	Malignant neoplasm without specification of site	Oncologic
126	Malposition; malpresentation	Elective
127	Medical examination/evaluation	Other
128	Melanomas of skin	Oncologic
129	Meningitis (except that caused by tuberculosis or sexually transmitted disease)	Acute
130	Menopausal disorders	Chronic
131	Menstrual disorders	Chronic
132	Miscellaneous mental health disorders	Chronic
133	Mood disorders	Chronic
134	Multiple myeloma	Oncologic

Supplementary file 1 – continued		
135	Multiple sclerosis	Chronic
136	Mycoses	Chronic
137	Nausea and vomiting	Chronic
138	Neoplasms of unspecified nature or uncertain behavior	Oncologic
139	Nephritis; nephrosis; renal sclerosis	Chronic
140	Nervous system congenital anomalies	Chronic
141	Non-Hodgkin`s lymphoma	Oncologic
142	Noninfectious gastroenteritis	Acute
143	Nonmalignant breast conditions	Elective
144	Nonspecific chest pain	Acute
145	Nutritional deficiencies	Chronic
146	Occlusion or stenosis of precerebral arteries	Other
147	Open wounds of extremities	Acute
148	Open wounds of head; neck; and trunk	Acute
149	Osteoarthritis	Elective
150	Osteoporosis	Chronic
151	Other acquired deformities	Elective
152	Other aftercare	Other
153	Other and ill-defined cerebrovascular disease	Acute
154	Other and ill-defined heart disease	Chronic
155	Other and unspecified benign neoplasm	Elective
156	Other bone disease and musculoskeletal deformities	Chronic
157	Other circulatory disease	Chronic
158	Other CNS infection and poliomyelitis	Chronic
159	Other complications of birth; puerperium affecting management of mother	Acute
160	Other complications of pregnancy	Elective
161	Other congenital anomalies	Chronic
162	Other connective tissue disease	Chronic
163	Other diseases of bladder and urethra	Acute
164	Other diseases of kidney and ureters	Chronic
165	Other diseases of veins and lymphatics	Chronic
166	Other disorders of stomach and duodenum	Chronic
167	Other ear and sense organ disorders	Chronic
168	Other endocrine disorders	Elective
169	Other eye disorders	Elective
170	Other female genital disorders	Chronic
171	Other fractures	Acute
172	Other gastrointestinal disorders	Chronic
173	Other hematologic conditions	Chronic
174	Other hereditary and degenerative nervous system conditions	Chronic
175	Other infections; including parasitic	Acute
176	Other inflammatory condition of skin	Chronic
177	Other injuries and conditions due to external causes	Acute
178	Other liver diseases	Chronic
179	Other lower respiratory disease	Chronic
180	Other male genital disorders	Elective
181	Other nervous system disorders	Chronic
182	Other non-epithelial cancer of skin	Oncologic
183	Other non-traumatic joint disorders	Chronic
184	Other nutritional; endocrine; and metabolic disorders	Chronic
185	Other perinatal conditions	Elective
186	Other pregnancy and delivery including normal	Elective
187	Other screening for suspected conditions (not mental disorders or infectious disease)	Elective
188	Other skin disorders	Chronic
189	Other upper respiratory disease	Chronic
190	Other upper respiratory infections	Elective
191	Otitis media and related conditions	Acute
192	Pancreatic disorders (not diabetes)	Acute
193	Paralysis	Chronic
194	Parkinson`s disease	Chronic
195	Pathological fracture	Chronic
196	Peri-; endo-; and myocarditis; cardiomyopathy (except that caused by tuberculosis or sexually transmitted disease)	Chronic
197	Peripheral and visceral atherosclerosis	Chronic
198	Peritonitis and intestinal abscess	Acute
199	Phlebitis; thrombophlebitis and thromboembolism	Acute
200	Pleurisy; pneumothorax; pulmonary collapse	Acute
201	Pneumonia (except that caused by tuberculosis or sexually transmitted disease)	Acute
202	Poisoning by nonmedicinal substances	Acute

**Supplementary file 1 – continued**		
203	Previous C-section	Other
204	Prolapse of female genital organs	Chronic
205	Pulmonary heart disease	Chronic
206	Regional enteritis and ulcerative colitis	Chronic
207	Rehabilitation care; fitting of prostheses; and adjustment of devices	Other
208	Residual codes; unclassified	Chronic
209	Respiratory distress syndrome	Other
210	Respiratory failure; insufficiency; arrest (adult)	Acute
211	Retinal detachments; defects; vascular occlusion; and retinopathy	Elective
212	Rheumatoid arthritis and related disease	Chronic
213	Schizophrenia and other psychotic disorders	Chronic
214	Screening and history of mental health and substance abuse codes	Other
215	Secondary malignancies	Oncologic
216	Septicemia (except in labor)	Acute
217	Sexually transmitted infections (not HIV or hepatitis)	Acute
218	Sickle cell anemia	Chronic
219	Skin and subcutaneous tissue infections	Acute
220	Skull and face fractures	Acute
221	Spinal cord injury	Acute
222	Spondylosis; intervertebral disc disorders; other back problems	Chronic
223	Sprains and strains	Acute
224	Substance-related disorders	Chronic
225	Superficial injury; contusion	Acute
226	Syncope	Acute
227	Systemic lupus erythematosus and connective tissue disorders	Chronic
228	Thyroid disorders	Chronic
229	Transient cerebral ischemia	Acute
230	Tuberculosis	Chronic
231	Urinary tract infections	Elective
232	Varicose veins of lower extremity	Elective
233	Viral infection	Acute

Supplementary file 2 – Definition of observed/expected ratios and exclusivity ratios

To characterize each cluster, in accordance with the research of Violán et al. (2019)[1] observed/expected $((O/\,E)_{xy})$ ratios and exclusivity (EX_{xy}) ratios were calculated for the medical specialties x within each cluster y. The $(O/E)_{xy}$ ratio is the observed prevalence of medical specialty x in cluster y (O_{xy}) divided by the expected prevalence of medical specialty x in the overall sample (E_x).

The observed prevalence was calculated by dividing the sum of the membership degrees M of cluster y for patients with medical specialty x by the sum of all the memberships degrees of cluster y. That is:

$$O_{xy} = \frac{\sum n \in S_x \, M_{ny}}{\sum_{n=1}^{N} M_{ny}}, \qquad (1)$$

where S_x is the set of all individuals with the medical specialty x, N is the total number of patients, and M_{ny} is the membership degree factor, corresponding to the membership of an individual n for cluster y. As an example, suppose a cluster consists of three patients. Two patients had a membership of 0.7 and 0.3, respectively, for this cluster and both these patients visited a dermatologist. The third patients had a membership of 0.5 for this cluster and this patient did not visit a dermatologist. In that case, the observed prevalence of the dermatologist for this cluster was 0.67, i.e. $\frac{0.7+0.3}{0.7+0.3+0.5} = 0.67$.

The expected prevalence of medical specialty x in cluster y is the prevalence of medical specialty x that we expect if their involvement would be unrelated to the clusters and therefore evenly divided over all clusters. Consequently, the expected prevalence of medical specialty x in cluster y is equal to the expected prevalence of medical specialty x in the overall sample (E_x). It is defined as:

$$E_x = \frac{n_x}{N}, \qquad (2)$$

where n_x is the number of patients with medical specialty x in the overall sample and N is the sample size.

Finally, the exclusivity ratio EX_{xy} is the ratio of the sum of the membership degrees of cluster *y* for patients with the specialty x and the total number of patients with the specialty involved (n_x). Hence:

$$EX_{xy} = \frac{\sum n \in S_x \, M_{ny}}{n_x}. \qquad (3)$$

In line with previous research, we considered a specialty to characterize a cluster when the O/E ratio was ≥2 or the exclusivity value was ≥25%[1-3].

References

1. Violán C, Foguet-Boreu Q, Fernández-Bertolín S, et al. Soft clustering using real-world data for the identification of multimorbidity patterns in an elderly population: cross-sectional study in a Mediterranean population. *BMJ Open*. 2019;9(8):e029594. doi:10.1136/bmjopen-2019-029594

2. Marengoni A, Roso-Llorach A, Vetrano DL, et al. Patterns of Multimorbidity in a Population-Based Cohort of Older People: Sociodemographic, Lifestyle, Clinical, and Functional Differences. *J Gerontol A Biol Sci Med Sci*. Mar 9 2020;75(4):798-805. doi:10.1093/gerona/glz137

3. Schäfer I, Kaduszkiewicz H, Wagner H-O, Schön G, Scherer M, van den Bussche H. Reducing complexity: a visualisation of multimorbidity by combining disease clusters and triads. *BMC Public Health*. 2014;14(1):1-14.

Supplementary file 3 – Diagnoses with a prevalence greater than 2% in the study population (n = 22133)

Diagnoses	Frequency(%)
Residual codes; unclassified	4039 (18.3)
Other nervous system disorders	3607 (16.3)
Other connective tissue disease	2488 (11.2)
Other non-epithelial cancer of skin	2475 (11.2)
Other circulatory disease	2419 (10.9)
Spondylosis; intervertebral disc disorders; other back problems	2271 (10.3)
Other skin disorders	2008 (9.1)
Other ear and sense organ disorders	1828 (8.3)
Other screening for suspected conditions (not mental disorders or infectious disease)	1731 (7.8)
Other upper respiratory disease	1520 (6.9)
Cataract	1476 (6.7)
Chronic obstructive pulmonary disease and bronchiectasis	1438 (6.5)
Asthma	1336 (6.0)
Cardiac dysrhythmias	1321 (6.0)
Osteoarthritis	1317 (6.0)
Other aftercare	1210 (5.5)
Other gastrointestinal disorders	1182 (5.3)
Other inflammatory condition of skin	1178 (5.3)
Other eye disorders	1175 (5.3)
Retinal detachments; defects; vascular occlusion; and retinopathy	1170 (5.3)
Other and unspecified benign neoplasm	1114 (5.0)
Other non-traumatic joint disorders	1083 (4.9)
Nonspecific chest pain	1064 (4.8)
Diabetes mellitus with complications	1040 (4.7)
Congestive heart failure; non-hypertensive	1004 (4.5)
Cancer of breast	996 (4.5)
Osteoporosis	948 (4.3)
Delirium dementia and amnestic and other cognitive disorders	861 (3.9)
Hyperplasia of prostate	845 (3.8)
Coronary atherosclerosis and other heart disease	835 (3.8)
Rehabilitation care; fitting of prostheses; and adjustment of devices	809 (3.7)
Abdominal pain	804 (3.6)
Glaucoma	798 (3.6)
Other female genital disorders	797 (3.6)
Chronic kidney disease	726 (3.3)
Malignant neoplasm without specification of site	708 (3.2)
Conditions associated with dizziness or vertigo	687 (3.1)
Essential hypertension	648 (2.9)
Rheumatoid arthritis and related disease	647 (2.9)
Cancer of prostate	630 (2.9)
Conduction disorders	598 (2.7)
Headache; including migraine	590 (2.7)
Menstrual disorders	587 (2.7)
Pneumonia (except that caused by tuberculosis or sexually transmitted disease)	576 (2.6)
Cancer of bronchus; lung	531 (2.4)
Heart valve disorders	521 (2.4)
Other lower respiratory disease	512 (2.3)
Genitourinary symptoms and ill-defined conditions	503 (2.3)
Thyroid disorders	484 (2.2)
Gastrointestinal hemorrhage	455 (2.1)
Prolapse of female genital organs	454 (2.1)
Cancer of rectum and anus	444 (2.0)

Supplementary file 4 - Optimal parameters for fuzzy c-means

To find the optimal cluster solution, we first identified the optimal parameters for the fuzzy c-means algorithm: the number of clusters (k) and the fuzziness parameter (m). Supplementary file 5 shows the behavior of all indices for all computed m and k values. Supplementary file 6 contains an enlargement of supplementary file 5, especially for the Xie-Beni index as it had large outliers for m=1.3-1.5. The partition coefficient and modified partition coefficient for m=1.4 and m=1.5 showed similar values and are therefore not fully separately visible in the plots. The optimal m parameter corresponds to the minimum value for the Xie-Beni index and partition entropy and the maximum value of the partition coefficient and silhouette index. The Xie-Beni index, partition coefficient, and partition entropy identified m=1.1 as optimal choice. The silhouette index with m=1.2 and m=1.3 showed slightly higher values compared to m=1.1, but overall m=1.1 showed the best values for all four indices, therefore m=1.1 was chosen as the optimal fuzziness parameter.

Supplementary file 7 shows the behavior of all indices with m=1.1 for all tested k-values. The optimal number of clusters (k) was the number for which the Xie-Beni and partition entropy had the lowest value and the partition coefficient and silhouette index had the highest value. The silhouette index and the partition coefficient identified k=6 as the optimal number of clusters, the partition entropy showed an optimal index at k=5 (followed by k=6). The Xie-Beni identified k=14 as the optimal number of clusters. We selected the mode of the identified optimal k's, k=6, as the optimal number of clusters for our analysis with the fuzziness parameter m=1.1.

Supplementary file 5 - Validation indices for m= 1.1, 1.2, 1.3, 1.4, 1.5

Behavior of all validation indices, for all tested m- and k-values (m = fuzziness-parameter, k = number of clusters). The minimum value for the Xie-Beni index (a) and partition entropy (c), and the maximum value of the partition coefficient (b) and silhouette index (c), correspond to the optimal m-parameter.

a – Xie Beni

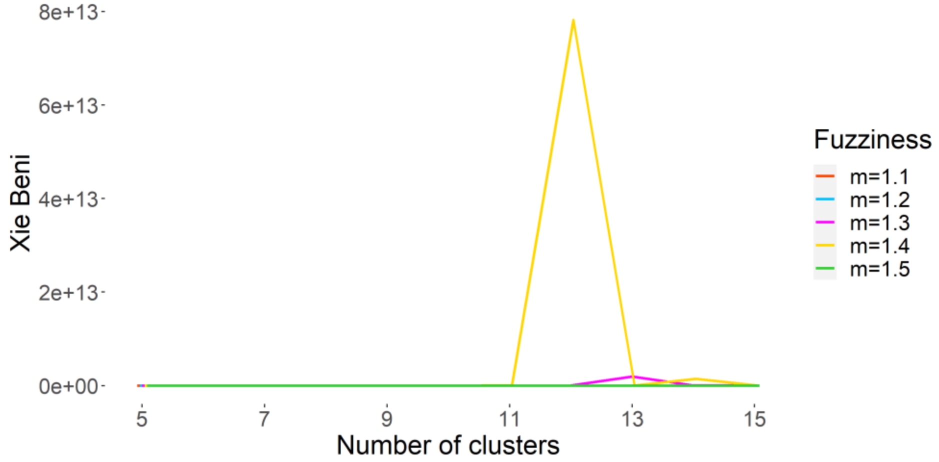

b – Partition Coefficient

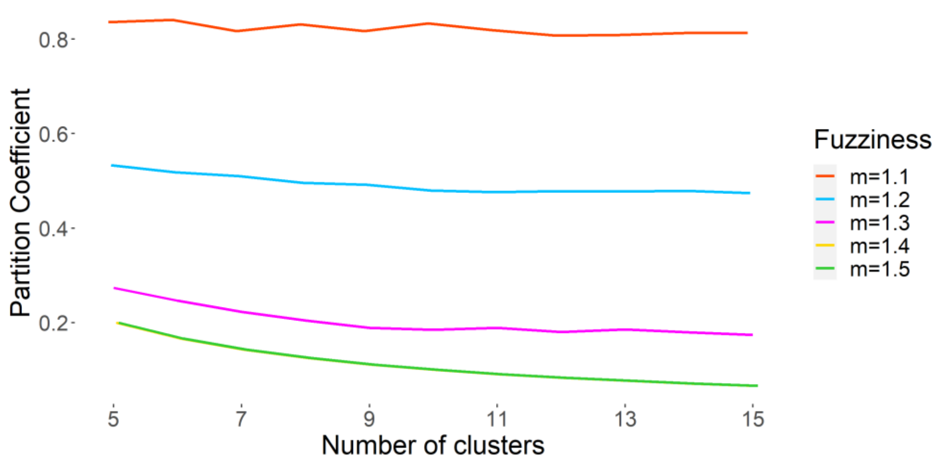

c – Partition Entropy

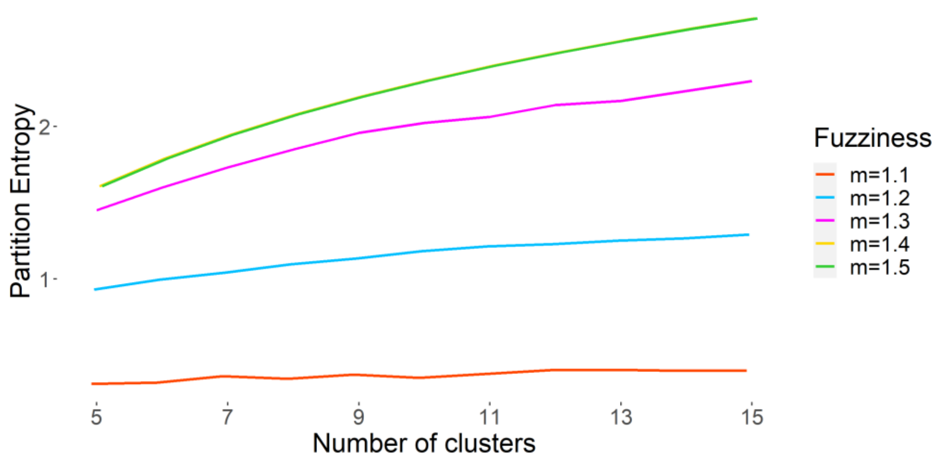

d – Silhouette Index

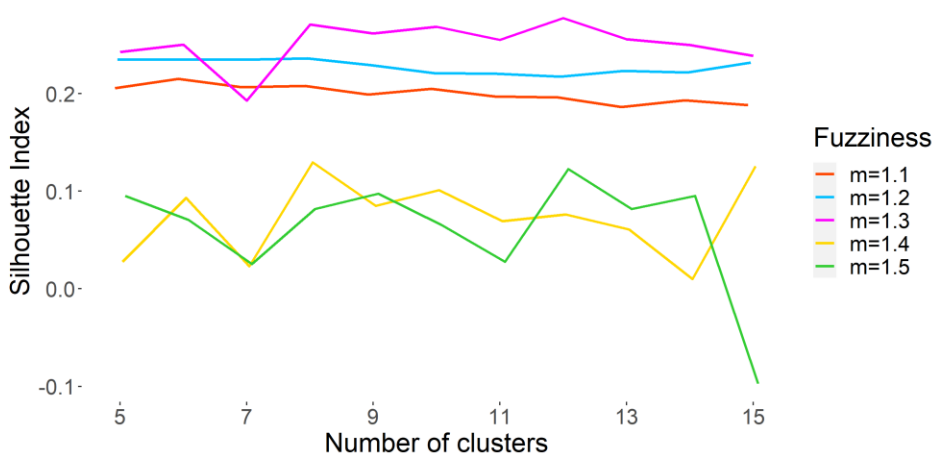

Supplementary file 6 - Validation indices for m= 1.1, 1.2, 1.5 (for Xie-Beni: only 1.1 & 1.2)

Behavior of all validation indices, with m= 1.1, 1.2 and 1.5 (Xie-Beni only 1.1 and 1.2) for all tested k-values (m = fuzziness-parameter, k = number of clusters). The minimum value for the Xie-Beni index (a) and partition entropy (c), and the maximum value of the partition coefficient (b) and silhouette index (c), correspond to the optimal m-parameter.

a – Xie Beni

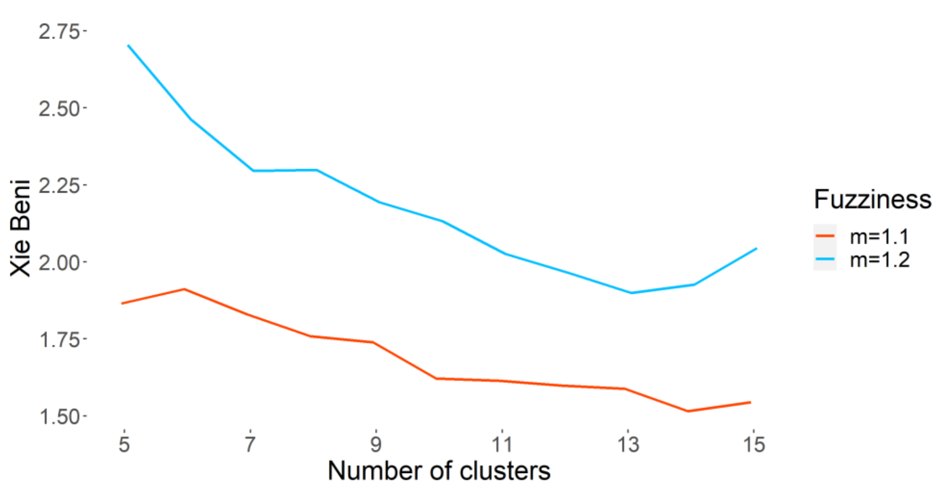

b – Partition Coefficient

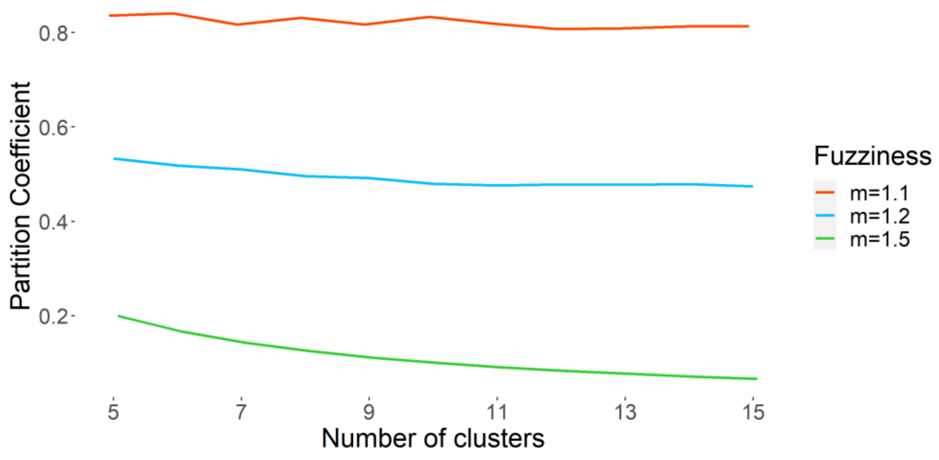

c – Partition Entropy

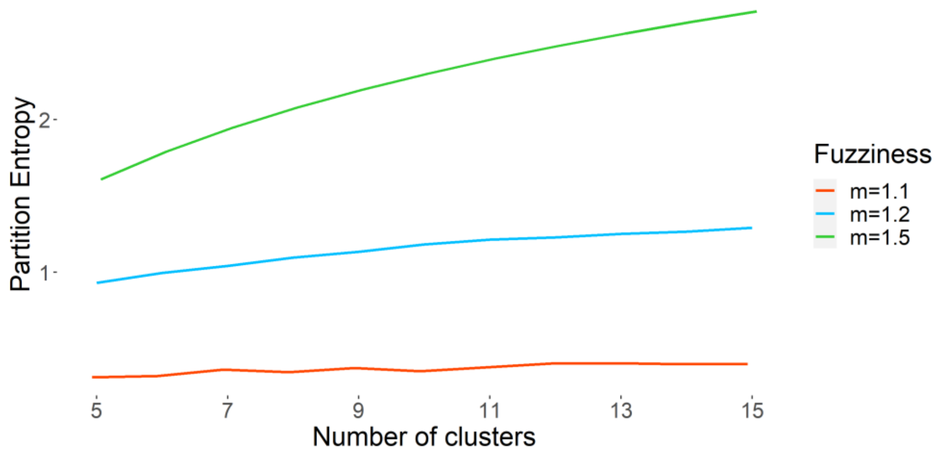

d – Silhouette Index

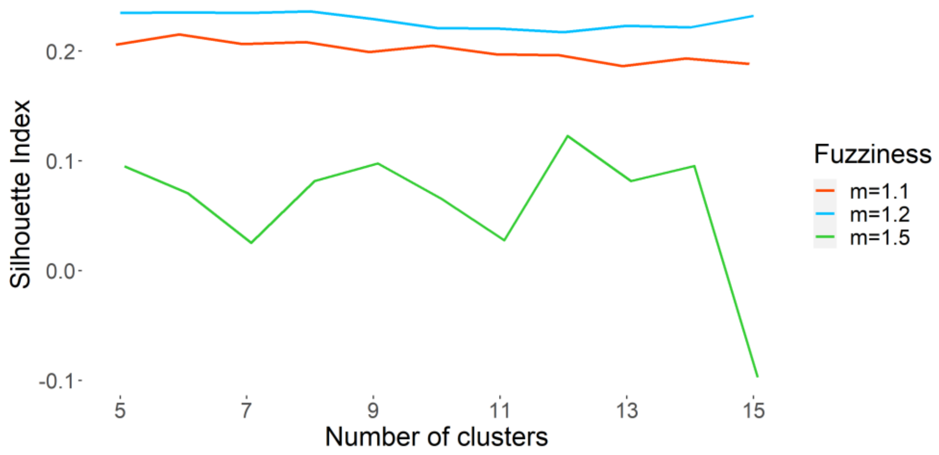

Supplementary file 7 - Validation indices for m= 1.1

Behavior of all validation indices, with m= 1.1 for all tested k-values (m = fuzziness-parameter, k = number of clusters). The minimum value for the Xie-Beni index (a) and partition entropy (c), and the maximum value of the partition coefficient (b) and silhouette index (c), correspond to the optimal m-parameter.

a – Xie Beni

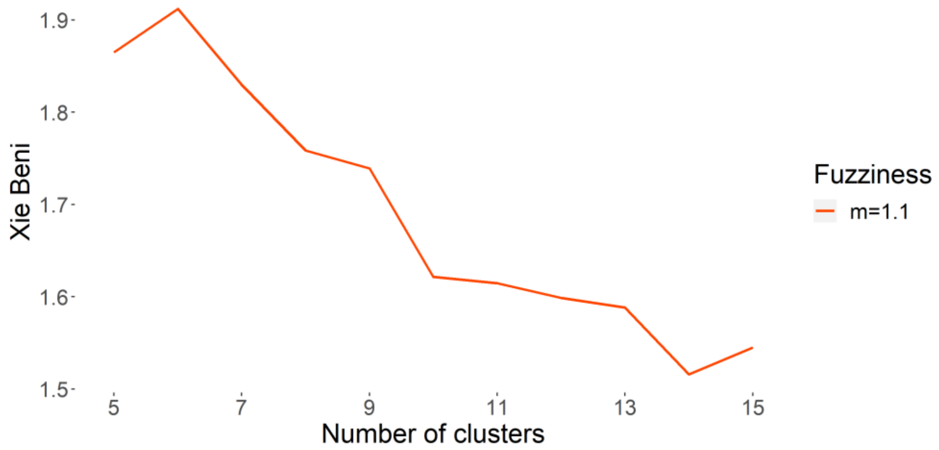

b – Partition Coefficient

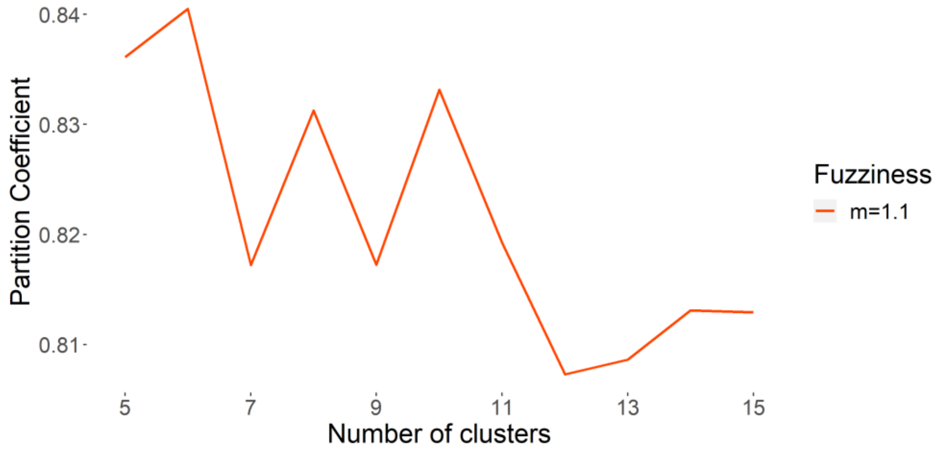

c – Partition Entropy

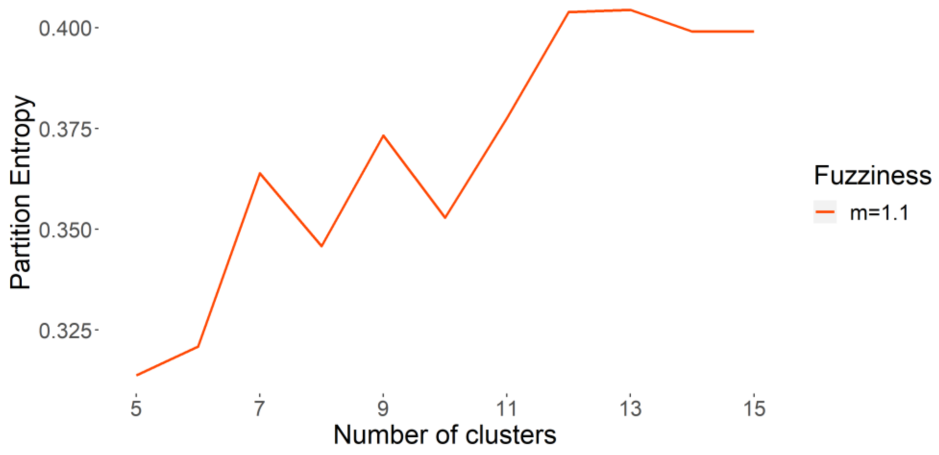

d – Silhouette Index

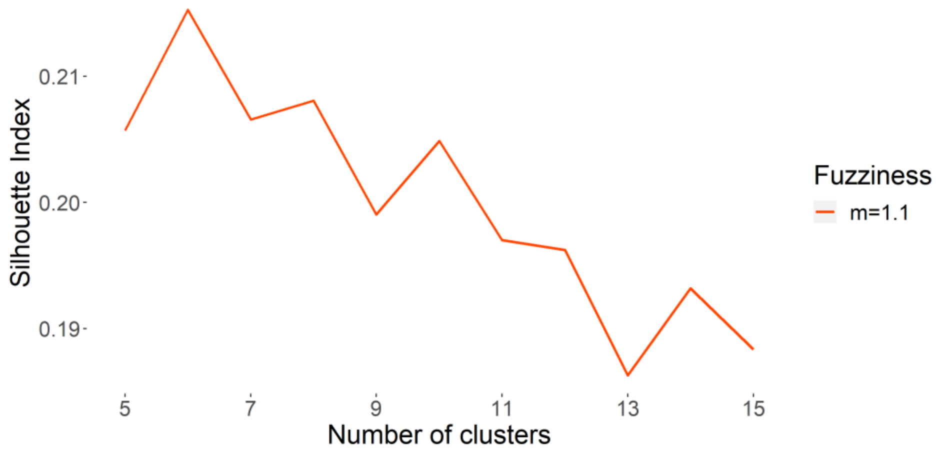

Supplement: Supplementary file 1 — Additional file 1: Supplementary file 1. Table of the 233 diagnosis groups used in this study from the Dutch Hospital Data-Clinical Classification Software (DHD-CCS). Supplementary file 2. Definition of observed/expected ratios and exclusivity ratios. Supplementary file 3. Diagnoses with a prevalence greater than 2% in the study population (n = 22133). Supplementary file 4. Optimal parameters for fuzzy c-means. Supplementary file 5. Validation indices for m= 1.1, 1.2, 1.3, 1.4, 1.5. Supplementary file 6. Validation indices for m= 1.1, 1.2, 1.5 (for Xie-Beni: only 1.1 & 1.2). Supplementary file 7. Validation indices for m= 1.1. [file 12913_2023_9961_MOESM1_ESM.pdf]
